# Inhibition of biofilm formation in *Mycobacterium smegmatis* by *Parinari curatellifolia* leaf extracts

**DOI:** 10.1186/s12906-017-1801-5

**Published:** 2017-05-30

**Authors:** Benjamin Bhunu, Ruvimbo Mautsa, Stanley Mukanganyama

**Affiliations:** Bio-molecular Interactions Analyses Group, Department of Biochemistry, University of Zimbabwe, P.O. Box MP 167, Mt. Pleasant, Harare, Zimbabwe

**Keywords:** *Parinari curatellifolia*, Biofilm formation, *Mycobacterium smegmatis*, Tuberculosis, Isoniazid, Kanamycin

## Abstract

**Background:**

Tuberculosis (TB) is a serious public health problem worldwide. *Mycobacterium tuberculosis* (*M. tuberculosis*) grows as drug tolerant pellicles. Agents that inhibit biofilm formation in *M. tuberculosis* have the potential to reduce the disease treatment period and improve the quality of tuberculosis chemotherapy. *Parinari curatellifolia* (*P. curatellifolia*) leaf extracts are claimed to treat symptoms similar to tuberculosis in ethnomedicinal practices. *Mycobacterium smegmatis* (*M. smegmatis*) is a surrogate organism used in antimycobacterial drug discovery assays. In this study, the effect of the leaf extracts of *P. curatellifolia* on *M. smegmatis* growth and biofilm formation was investigated in order to determine the basis of its use in traditional medicinal use.

**Methods:**

Phytochemicals from *P. curatellifolia* leaves were prepared using a mixture of 50% dichloromethane (DCM): 50% methanol and by serial exhaustive extraction using different solvents of decreasing polarity. The solvents were used in the following order, hexane > dichloromethane > ethyl acetate > acetone >ethanol > methanol > water. The micro-broth dilution method was used as an antimycobacterial susceptibility test to screen for the extract that effectively inhibited *M. smegmatis* growth and biofilm formation. Biofilm quantification was performed by staining the biofilms with crystal violet and determining the amount of the stain using a spectrophotometer. In addition, the effects of combining the most active extract with kanamycin were also investigated.

**Results:**

The minimum inhibitory concentrations (MIC) of the extracts were found to be 6.2 μg/ml for the acetone extract, 12.5 μg/ml for both the ethanol and the total extract and 50 μg/ml for both the methanol and ethyl acetate extracts. The ethanol extract, dichloromethane extract and water extract were the only extracts that effectively inhibited biofilm formation in *M. smegmatis*. Combining the ethanol extract with kanamycin enhanced the effect of the ethanol extract in terms of inhibition of biofilm formation.

**Conclusions:**

*P. curatellifolia* leaves contain phytochemicals that have the potential to be used both as antimycobacterial and anti-biofilm formation compounds.

## Background

Despite current improvements in the diagnosis and treatment of tuberculosis, the disease remains a serious public health problem globally [1]. In the year 2013, there were approximately 9 million new cases of tuberculosis (TB) and 1.5 million deaths caused by the infection [2]. Countries experiencing the highest burden of tuberculosis are from the developing world, especially in Africa and Asia [3]. Some of the factors responsible for the increasing incidence of tuberculosis include the emergence of the HIV (human immunodeficiency virus) pandemic, the deterioration of public health systems in the developing world and the evolution of drug resistant strains of tuberculosis [4, 5].

The development of drug resistant phenotypes of tuberculosis is one of the major obstacles hampering effective control of the disease [6]. Isolates that are resistant to one or more anti-tuberculosis drugs have been identified [5]. In addition, extensively drug-resistant (XDR), and totally drug-resistant (TDR) *M. tuberculosis* strains have been reported [7]. The existence of drug-resistant MTB phenotypes in an infection extends the treatment period to 20 months and requires the use of sometimes toxic, expensive and less efficient second line anti-TB drugs [5]. In most cases, the chances of successfully treating XDR-TB and TDR-TB are often very low [8]. There is, therefore, an urgent need to introduce new sources of drugs for the treatment of tuberculosis in order to overcome the problem of drug resistance and achieve eradication of the disease [7].

Currently, the main hurdle in TB research is poor understanding of the mechanisms by which MTB evades both the host immune response and antibiotic treatment. In addition, there is poor understanding of the mechanisms used by the pathogen to form persistent infections that exhibit increased drug tolerance [9]. During the early stages of tuberculosis infection, the mycobacterium evades the host immune response and antibiotic action by infecting and hiding within infected host cells such as macrophages [10]. Stress factors such as low oxygen and nutrient concentrations may promote some of the mycobacteria to become dormant and grow into structures that closely resemble biofilms [11].

A number of virulence factors have been discovered that assist the survival of *M. tuberculosis* inside the human host [12]. Approximately 99% of microbes living in the biosphere are capable of forming multi-cellular communities called biofilms. These are structured communities of sessile bacterial cells embedded within a self-produced matrix. Biofilm matrices are made up of proteins, DNA and polysaccharides that together act as physical and physiological barriers to antimicrobial agents produced by the host immune response or by antibiotics [13]. Studies suggest that biofilms are important in establishing chronic, persistent, drug tolerant infections in most pathogenic and opportunistic bacterial infections [14]. Biofilms thus provide powerful physical barriers to penetration of antibiotics and nutrients [15].

The distribution of *Parinari curatellifolia*, (*Chrysobalanaceae* family), is wide spread throughout Africa. This tree has been used as a source of ethnomedicinal extracts in Africa [16]. The traditional uses of the plant include treatment of inflammation, cancer, wounds and bacterial infections and dressing of fractures and dislocations [17]. Other conditions that are claimed to be treated by the plant include, fever, typhoid, and malaria. The plant has also been reported to be effective in treating symptoms similar to tuberculosis [18]. The ability of plant and plant extracts to treat diseases is largely attributed to the presence of phytochemicals. Phytochemicals within the plants show different therapeutic effects in the treatment of various chronic diseases [19]. Plant secondary metabolites also have antiviral and antimicrobial activity. In a study by Tochukwu and Usman [20], a crude extract of *P. curatellifolia* contained the following secondary metabolites; saponins, alkaloids, flavonoids, steroids, tannins, and cardiac glycosides. Other studies screening the phytochemical constituents of *P. curatellifolia* showed similar results and also showed the presence of terpenoids [21]. *M. smegmatis* is a non-pathogenic mycobacterium and has a drug sensitivity profile comparable to *M. tuberculosis* [22]. This mycobacterial species can be used in preliminary studies to select compounds with potential activity against *M. tuberculosis.* Screening extracts against *M. tuberculosis* can be an aseptically rigorous procedure owing to its extremely infective nature. Thus, a biological safety level 3 (BSL3) cabinet has to be used when handling *M. tuberculosis* [23]. *M. tuberculosis* also grows slowly, with a generation time of 16–24 h [24]. The slow growing and highly infectious nature of *M. tuberculosis* has slowed down the discovery of new anti-TB agents [25]. In this study, we used the non-infective *M. smegmatis* because of its fast growing nature and basic similarities with *M. tuberculosis*. Other researchers studying biofilms in mycobacteria have also used *M. smegmatis* as one of their model organisms [26]. The aim of this study, therefore, was to investigate the antimycobacterial activity of the leaf extracts of *P. curatellifolia* against growth and biofilm formation in *M. smegmatis.*

## Methods

### Bacterial strains and reagents

The bacterium used in this study was the *M. smegmatis* 155 mc^2^ laboratory strain. It was obtained from Professor Daniel Steenkamp, Department of Clinical Laboratory Sciences, University of Cape Town. Dimethyl sulfoxide (DMSO), Middlebrook 7H9 and Casein hydrosylate were obtained from Sigma Aldrich (Steinheim, Germany). All the reagents used in this study were high grade chemicals obtained from various sources.

### Plant collection

*P. curatellifolia* fresh leaves were collected from Centenary (Latitude 16^o^48’00″S, Longitude 31^o^07’00″E, elevation above sea level 1156 m) in Mashonaland Central Province of Zimbabwe in the summer period of February 2015. The identity of the plant samples was authenticated by Mr. Christopher Chapano, a taxonomist at the National Herbarium located at the Harare Botanical Gardens, Harare, Zimbabwe. Voucher specimens (C6E7) were made and stored in the Biomolecular Interactions Analyses Laboratory at the Department of Biochemistry, University of Zimbabwe, Harare, Zimbabwe and at the National Herbarium. The leaves were washed with distilled water and then dried in an oven at 40 ˚C. The leaf powder was prepared using a blender (Philips Co., Shanghai, China). The leaf powder was stored in closed containers at room temperature.

### Preparation of plant extracts

Phytochemicals from 50 g of powdered *P. curatellifolia* leaf material were extracted by maceration using 500 ml of a solution of 50%:50% dichloromethane (DCM) and methanol respectively for the total extraction. The mixture was filtered using Whatman No.1 paper. The leaf debris was discarded while the filtrate was left in a sterile fume hood to allow the solvent to evaporate and to completely dry the extract. The amount of extract obtained was measured using an analytical balance and recorded as percentage yield.

Serial exhaustive extraction was carried out sequentially using different solvents of decreasing polarity on the same leaf sample. The serial exhaustive extraction was performed in the following sequence: hexane extraction > DCM extraction > acetone extraction > ethyl acetate extraction > ethanol extraction > methanol extraction > water extraction. At each extraction stage, the leaf debris generated after filtration was left to dry in a sterile fume hood before being used in the subsequent extraction stage. The solvent dissolving the extracts was evaporated and the extracts were left to dry in a sterile fume hood. The mass of each extract was then determined.

### Growth of *M. smegmatis*

*M. smegmatis* was grown in Middlebrook 7H9 media (5.2 g/L) supplemented with 1 g/L casein acid hydrolysate. The media components were dissolved in 40 ml of boiled distilled water and sterilised by autoclaving. A single colony of *M. smegmatis* was inoculated into 20 mL of media in a 50 ml centrifuge tube and incubated with an appropriate negative control. The tubes were incubated overnight at 37 ˚C with constant shaking at 120 rpm in a Lab Companion incubator (Jeio Tech, Korea).

### The effect of *P. curatellifolia* leaf extracts on *M. smegmatis* growth

The effects of isoniazid, kanamycin and the eight *P. curatellifolia* leaf extracts obtained from the serial exhaustive and total extraction were investigated for their growth inhibitory effect using the microbroth dilution method of Vipra et al., [27]. Isoniazid was dissolved in DMSO to give a solution containing a final concentration of 5% DMSO and 200 μg/ml isoniazid. The dissolved drug was serially diluted in a 2-fold manner with media containing 5% DMSO up to a concentration of 0.8 μg/ml isoniazid. The different concentrations of isoniazid at 100 μl each were placed into a 96-well micro-titre plate (Greiner-bione, Sigma- Aldrich, St. Louis, MO, USA). Exponentially growing *M. smegmatis* culture was standardised using 0.5 McFarland’s standard solution to give a cell suspension of 2 × 10^6^ cfu/ml. A volume of 100 μl of the standardised cell suspension was then placed into each well of the 96- well microplate. Determination of cell densitypre-incubation was carried out using a Tecan microplate reader (Tecan Genios-Pro, GrÖdig, Austria) at 590 nm. The plate was then incubated for 24 h at 37 ˚C in a Lab Companion incubator (Jeio Tech, Korea) without shaking. After the incubation period, cell density was again measured at 590 nm. In order to visualise viable cell growth on the plate, 25 μl of 3-(4, 5-dimethylthiazol-2)-2, 5-diphenyltetrazolium bromide (MTT) solution (1 mg/ml) was added to each well of the microtitre plate containing 200 μl of solution. This was followed by an incubation period of 45 min and a reading of the absorbance of the MTT was taken at 590 nm. A graph of optical density at 590 nm against isoniazid concentration was then plotted to determine the MIC. The same procedure was carried out to determine the MIC of kanamycin and the plant extracts.

### Screening for an extract that effectively inhibits biofilm formation in *M. smegmatis*

Biofilm formation in *M. smegmatis* was studied using a method developed by Hawser et al.*,* [28] with some modifications. Polyvinyl chloride (PVC) discs of surface area 1 cm^2^ were cut from PVC pipes and the disks were sterilized by placing them in 70% alcohol. Three sterilized disks were placed at equidistant positions in each well of a sterile 6-well Nunclon™ (Sigma-Aldrich, Steinheim, Germany) plate. A 24-h old *M. smegmatis* cell culture was centrifuged at 3500 rpm for 3 min in a Hettich Rotofix 32 centrifuge (Tuttlingen, Germany). The supernatant of the centrifuged cell culture was discarded and 10 ml of 0.9% sodium chloride solution was used to wash the cells. The cell suspension was centrifuged (3500 rpm) for 3 min, the supernatant discarded and 5 ml of sterile media added before standardisation with 0.5 McFarland standard solution to give a cell suspension of 5 × 10^8^ cfu/ml. A volume of 5 ml of the cells were dispensed into each well of the 6-well plates containing the discs. The plates were incubated at 37 ^˚^C for 2 h to allow the cells to adhere to the discs. The non-adherent cells were removed by gentle washing with 5 ml of 0.15 M phosphate buffered saline (PBS) pH 7.2. The discs were removed from the 6-well plate and each disc was placed singly into each well of a 12-well tissue culture plate containing 3 ml of 100 μg/ml *P. curatellifolia* leaf extract sample. The extracts, the positive control (kanamycin) and the negative control (DMSO) samples were placed in duplicate wells. Another single well containing the PVC disks and no cells served as the sterility control. The plates were incubated for 72 h at 37 ˚ C with shaking (30 rpm). After incubation, the media was removed using a Pasteur pipette and the wells were washed five times with PBS. The biofilms formed were stained by exposing the PVC disks to 4 ml of 0.1% crystal violet solution and incubating at room temperature for 45 min. The crystal violet was removed by washing three times with distilled water and the plates were left to dry completely. The crystal violet, bound to the biofilms on the discs, was dissolved in 4 mls of 95% ethanol and its absorbance was measured at 590 nm using a Tecan microplate reader (Tecan Genios-Pro, Grödig, Austria). The absorbance values were used to plot a graph of biofilm formation versus plant extract concentration. Since the ethanol extract was found to be the most effective at inhibition of biofilm formation, an assay to determine the most effective concentration to inhibit biofilm formation using concentrations of 0–1000 μg/ml was also performed.

### The effect of combining the ethanol extract with kanamycin on biofilm formation

Biofilm formation was carried out as before but with some modifications. The effect of varying kanamycin concentration from its MIC value of 1.6 μg/ml to a quarter of the MIC value whilst keeping the ethanol plant extract concentration constant at 100 μg/ml was investigated. In addition, the effect of varying the ethanol plant extract concentration from 100 μg/ml to 12.5 μg/ml whilst keeping the kanamycin concentration at 1.6 μg/ml was determined.

### Statistical analyses

Statistically significant differences between the mean of the controls and the tests were analysed using one way ANOVA with Dunnett’s multiple comparison post-test. The analyses were carried using the software GraphPad Prism 6 (Version 6.03 GraphPad Software Inc. San Diego, California U.S.A).

## Results

### Extraction of phytochemicals from *P. curatellifolia* leaves

In general, the serial exhaustive extraction method produced a higher amount of plant extract of 9.1 mg combined mass when compared to the total extraction method that produced 6. 13 mg. In the serial exhaustive extraction, the following masses were obtained for the different solvent extracts; hexane - 0.5 mg, DCM - 0.42 mg, acetone - 2.53 mg, ethyl acetate - 0.19 mg, ethanol - 0.77 mg, methanol - 3.23 mg and water - 1.45 mg. The total percentage yield of plant extract obtained from the serial exhaustive extraction was 18.19% compared to 12.25% using the total extraction method.

### The effect of *P. curatellifolia* leaf extracts on *M. smegmatis* growth

**Fig. 1:**
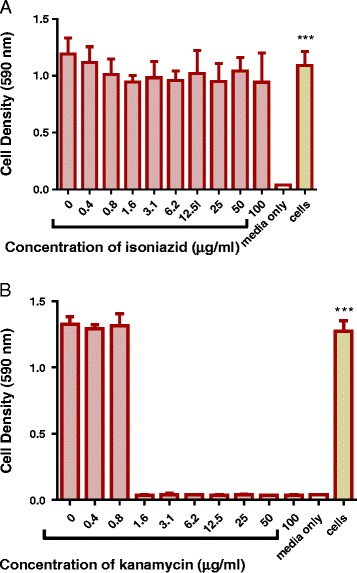
The effect of standard antimycobacterial agents on the growth of *M. smegmatis*. *M. smegmatis* mc^2^ 155 was treated with different concentrations of isoniazid (**a**) and kanamycin (**b**) from 0 μg/ml to 100 μg/ml. The bacteria were incubated with drugs at 37 ˚C for 20 h and cell density was determined by measuring absorbance of reduced MTT at 590 nm

**Fig. 2:**
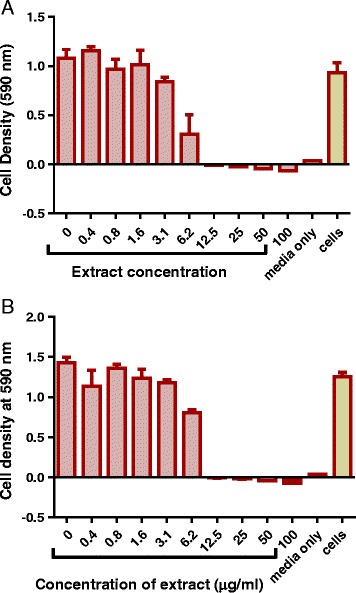
The effect of *P. curatellifolia* acetone and ethanol extracts on the growth of *M. smegmatis*. *M. smegmatis* mc^2^ 155 laboratory strain at concentration of 2 × 10^6^ cfu/mL was treated with different concentrations of the acetone (**a**) and ethanol extract (**b**) from 0 μg/ml up to 100 μg/ml. The bacteria were incubated with extracts at 37 ˚ C for 20 h and cell density was determined by measuring absorbance of reduced MTT at 590 nm. The error bars on the graphs indicate the standard deviation from mean (*n* = 4). Dunnet’s multiple comparison test was used to compare columns using Graphpad prism 6 software. The asterisks (*) indicate statistical significant differences with the sterility control (media) * < 0.05 *** < 0.0001

**Fig. 3:**
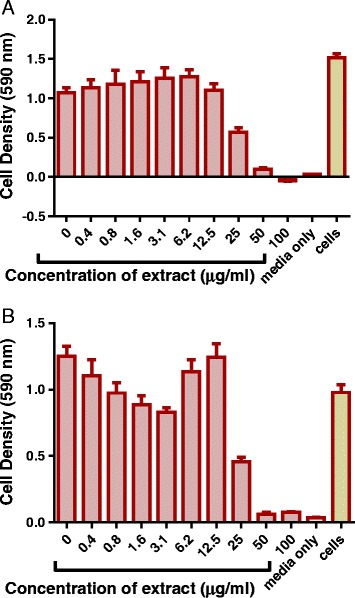
The eeffects of *P. curatellifolia* ethyl acetate fraction and the methanol extract on *M. smegmatis* growth. The *M. smegmatis* mc^2^ 155 laboratory strain at a concentration of 2 × 10^6^ cfu/mL was treated with different concentrations of the ethyl acetate (**a**) and methanol (**b**) extracts. The bacteria were incubated with extracts at 37 ˚ C for 20 h and cell density was determined by measuring absorbance of reduced MTT at 590 nm. The error bars on the graphs indicates the standard deviation from the mean (*n* = 4). Dunnet’s multiple comparison test was used to compare columns using Graph pad prism 6 software. The asterisks (*) indicate statistical significant differences with the sterility control (media) * < 0.05 *** < 0.0001

**Fig. 4:**
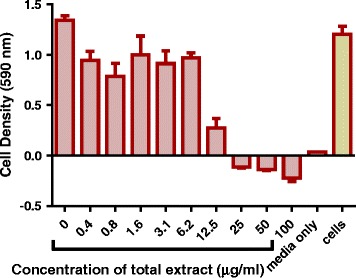
The effects of *P. curatellifolia* total extract on *M. smegmatis* growth. The *M. smegmatis* mc^2^ 155 laboratory strain at a concentration of 2 × 10^6^ cfu/mL was treated with the total extract from concentration of 0 μg/ml to 100 μg/ml of the plant extract. The bacteria were incubated with extracts at 37 ˚ C for 20 h and cell density was determined by measuring absorbance of reduced MTT at 590 nm. The error bars on the graphs indicate the standard deviation from mean (*n* = 4). Dunnet’s multiple comparison test was used to compare the columns using graph pad prism 5 software. The asterisks (*) indicate statistical significant differences with the sterility control (media) * < 0.05 *** 0.0001

Kanamycin had a minimum inhibitory concentration (MIC) value of 1.6 μg/ml when tested against *M. smegmatis*. Kanamycin was used as the positive control drug as the strain of *M. smegmatis* used in this study did not respond to 100 μg/ml of isoniazid (Fig. 1). Of all the eight extracts that were investigated for their effects on growth, the acetone extract was the most effective in the inhibition of growth of *M. smegmatis* and produced a MIC of 6.2 μg/ml. Both the ethanol extract and the total extractproduced a MIC of 12.5 μg/ml. Similar growth inhibitory activities were observed for the methanol extract and ethyl acetate extract (MICs of 50 μg/ml). The water extract, hexane extract and DCM extract exhibited no activity against *M. smegmatis* (Figs. 2, 3 and 4).

### Screening for an effective *P. curatellifolia* leaf extract that inhibited *M. smegmatis* biofilm formation

**Fig. 5:**
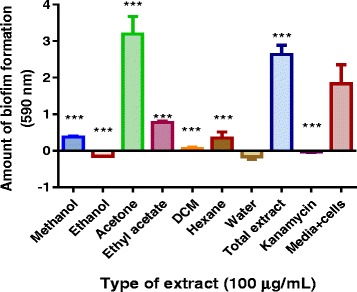
The effects of 100 μg/ml of the different P*. curatellifolia* leaf extracts on *M. smegmatis* biofilm formation. *M. smegmatis* was incubated with the plant extracts for 72 h at 37 ˚ C. The biofilm mass that formed during the incubation period was stained using crystal violet. The absorbance values of crystal violet are directly proportional to the amount of biofilm that had formed. The dye was extracted using ethanol and quantified spectrophotometrically at 590 nm

**Fig. 6:**
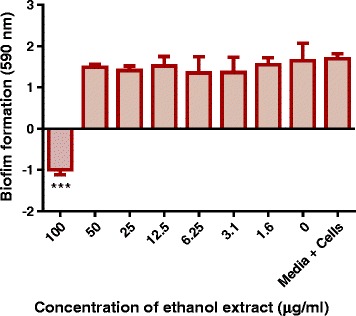
The effect of the different concentrations of the ethanol extract on *M. smegmatis* biofilm formation. *M. smegmatis* was incubated in different concentrations of the ethanol extract of *P. curatellifolia* leaf powder for 72 h at 37 ˚ C. The biofilm mass that formed during the incubation period was stained using crystal violet staining which was quantified spectrophotometrically at 590 nm. The error bars indicates the standard deviation from mean (*n* = 4). Dunnet’s multiple comparison test was used to compare columns using graph pad prism 5 software. The asterisks (*) indicate statistical significant differences with the growth control (media + cells) * < 0.05 *** < 0.0001

Kanamycin, water, DCM and ethanol leaf extracts inhibited biofilm formation in *M. smegmatis* at the tested concentration of 100 μg/ml (Fig. 5)*.* The ethyl acetate extract and hexane extract had significant anti-biofilm activities however both extracts failed to completely inhibit *M. smegmatis* biofilm formation. Compared to the control, the acetone extract and the total extract promoted, rather than, inhibited biofilm formation in *M. smegmatis*. The ethanol extract was found to be the most significant at inhibiting biofilm formation at 100 μg/ml concentration. Therefore, lower concentrations of ethanol extract, using a 2 fold serial dilution from 100 to 0 μg/ml, were also tested,. However, these lesser concentrations did not significantly inhibit biofilm formation (Fig. 6).

### The effects of combining the ethanol extract with kanamycin on *M. smegmatis* biofilm formation

**Fig. 7:**
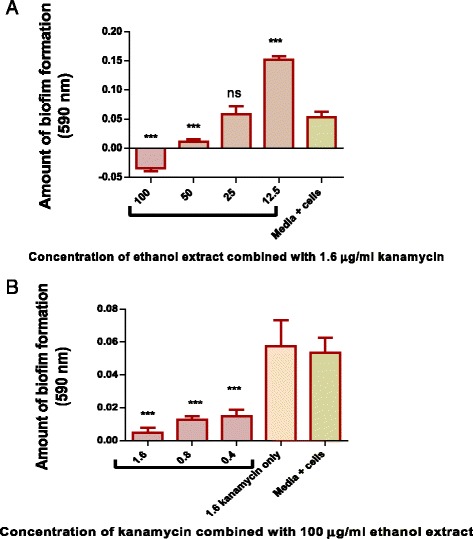
The effect of combining *P. curatellifolia* ethanol extract with kanamycin. **a** *M. smegmatis* was incubated in a medium containing 1.6 μg/ml kanamycin and varying ethanol extract concentration for 72 h. The amount of biofilm that had formed was determined by staining the biofilms with crystal violet, dissolving the crystal violet on the stained biofilms and spectrophotometrically measuring the amount of dye. **b** *M. smegmatis* was incubated for 72 h in a medium containing 100 μg/ml ethanol extract and varying concentration of kanamycin from MIC value to quarter MIC value. The Error bars indicates the standard deviation from mean (*n* = 4). Dunnet’s multiple comparison test was used to compare columns using graph pad prism 5 software. The asterisks (*) indicate statistical significant differences with the growth control (media + cells) * < 0.05 *** < 0.0001

The effect of kanamycin, at its MIC of 1.6 μg/ml, on *M. smegmatis* biofilm formation, was investigated and the drug did not inhibit biofilm formation. *M. smegmatis* biofilm formation at constant ethanol extract concentration (100 μg/ml) and varying kanamycin concentration, from the MIC value (1.6 μg/ml) to 0.25 x MIC value, was also investigated. The combination of kanamycin at 1 x MIC, 0.5 x MIC and 0.25 x MIC concentrations with the plant extract at a constant concentration of 100 μg/ml enhanced biofilm formation. The effectiveness of the ethanol extract at inhibiting biofilm formation was also enhanced by combining the different ethanol extracts concentration with a constant concentration of kanamycin at 1.6 μg/ml. However, at a concentration of 12.5 μg/ml, the ethanol extract combined with kanamycin promoted biofilm formation (Fig. 7).

## Discussion

The objective of this study was to investigate the effects of *P. curatellifolia* leaf extracts on *M. smegmatis* biofilm formation. The effect of the extract on biofilm formation was determined after initially assessing whether the extracts inhibited the growth of the cells. *M. smegmatis* is a surrogate mycobacterium used in preliminary anti-tubercular drug discovery assays [29]. The key aspects of *M. tuberculosis* biology are reminiscent of biofilm behaviour, for example, the ability of tuberculosis to grow in cell culture as bacterial clumps called cords, which are a correlate of virulence [30]. *M. tuberculosis* is able to effectively survive inside the human host and produce persistent infections despite rigorous drug treatments and the presence of an active immune response [31]. In addition, the treatment of tuberculosis takes a long period, ranging between six to nine months and the infection is often characterised by relapsing infections after discontinuing drug therapy [32]. In humans, clumps of *M. tuberculosis* contained in an acellular matrix have been found. The clumps were located in certain caveating lesions that were undergoing liquefaction in the human lungs [33]. All these characteristics of Mtb are reminiscent of an organism that is able to produce biofilms.

Biofilm formation by various pathogenic organisms has been demonstrated to be tolerant to elevated levels of various antimicrobials [15, 33]. Ojha et al.*,* [14] showed that *M. tuberculosis* grows in structures similar to biofilms called pellicles and these colonies were found to be drug-resistant. In addition, in the same study, tuberculosis pellicles were shown to confer drug resistance to drug susceptible *M. tuberculosis* mutants. Therefore, targeting *M. tuberculosis* biofilms is a potential way of improving TB chemotherapy.

*P. curatellifolia* is claimed to treat symptoms similar to tuberculosis [18, 34]. There have been previous studies conducted to investigate the anti-mycobacterial properties of the plant and these confirmed the antimicrobial activity of the plant extracts against *M. aurum* [35]. *P. curatellifolia* has also been shown to have antibacterial activity against a variety of bacterial species [36]. Phytochemical analysis of *P. curatellifolia* plant leaves has demonstrated the presence of saponins, steroids, alkaloids tannins, flavonoids and cardiac glycosides [37]. The presence of these secondary metabolites in the plant leaves indicate the potential use of the plant as a source of antimicrobial agents. The percentage yield of *P. curatellifolia* leaf extract from the total extraction and serial exhaustive extraction was 12.25% and 18.19% respectively. The percentage yield results suggest that the serial exhaustive extraction is a better extraction method than the total extraction (50% DCM: 50% Methanol) since more mass of the extract was obtained from the serial exhaustive extraction compared to the total extraction. The serial exhaustive extraction method used solvents with varying polarities to extract phytochemicals from the plant. In contrast, in the total extraction method, the solvent used was an immiscible mixture of methanol and DCM. Methanol is highly polar, while DCM is non-polar [38]. From the serial exhaustive extraction it was noted that more phytochemicals were extracted using polar solvents than non-polar solvents. Methanol was the most effective solvent for extraction followed by acetone and then water. These three solvents are polar in nature; water and methanol extract only polar phytochemicals, while acetone extracts both non polar and polar phytochemicals [39]. The majority of phytochemicals in *P. curatellifolia* leaves are polar compounds since phytochemicals are extracted more with solvents of a more polar nature.

Kanamycin is a second-line tuberculosis drug [40]. The use of kanamycin as a positive control in this study arose as a result of the insensitivity that was displayed by *M. smegmatis* towards isoniazid. The MIC value for kanamycin was found to be 1.6 μg/ml. In a study conducted by Taniguchi et al.*,* [41], aimed at analysing the molecular resistance to kanamycin in *M. smegmatis*, the MIC value for the wild type *M. smegmatis* was found to be 5 μg/ml. In another study done by Danilchanka et al., [42], designed to investigate the role of porins in the uptake of antibiotics by *M. smegmatis*, the wild type *M. smegmatis* was found to have an MIC value of 1.25 μg/ml. The MIC values obtained in this study are thus comparable with values obtained in other reports.

Of the eight extracts studied, the acetone extract was the most effective extract at inhibiting the growth of *M. smegmatis*. When the acetone extract was shaken, it produced froth indicating the presence of saponins [16]. Acetone is known to extract both hydrophilic and hydrophobic phytochemicals [39] and it is likely that the acetone extract contained a higher number of phytochemical species than the other extracts by the virtue of the mass obtained. The other extracts from *P. curatellifolia* leaves such as the ethanol extract, total extract, methanol and ethyl acetate also had significant bioactivities against *M. smegmatis*, although these activities were less than that of the acetone extract. The existence of more than one plant extract which was active against *M. smegmatis* suggests that *P. curatellifolia* leaves are a rich source of bioactive compounds that have the potential to lead to discoveries of new anti-mycobacterial compounds.

The activity produced by the extracts could be due to the presence of individual phytochemicals which are active against *M. smegmatis* or could be as a result of the synergistic effect of two or more phytochemicals that are contained in the extracts [43]. Isolation and purification of each of the individual phytochemicals that are contained in the extracts is required to determine if the bioactivity that was produced by the extracts is a result of a single phytochemical or if it was as a result of the combined effect of several phytochemicals. If the activity exhibited by the extracts was as a result of single compounds, those compounds may be used as candidates for new anti-mycobacterial drug discovery.

The water, DCM and ethanol extracts were found to be effective at inhibiting biofilm formation in *M. smegmatis*. However, the DCM and water extract had no appreciable bioactivities against the growth of *M. smegmatis*. Traditional healers use water as a solvent to extract phytochemicals from plant materials when treating different kinds of illness Sasidharan et al., [44]. In most phytochemical studies, the water extracts of different plants are reported to have no bioactivities [45]. Results from this study suggest that the water extract does not necessarily kill microbes but, rather, the extract prevents the clumping together of microbes. The clumping of microbes during the course of an infection leads to formation of microbial biofilms. When microbes are prevented from forming biofilms, they are eft exposed to the vast immune intervention mechanisms produced by the host against the pathogens [14].

Despite having an excellent anti-mycobacterial activity, the acetone extract, failed to inhibit *M. smegmatis* biofilm formation but rather promoted it at the tested concentration of 100 μg/ml. The acetone extract might have contained antibacterial phytochemicals that stimulate a stress response in the bacteria. Several studies have demonstrated that when microbes are stressed, such as under antibiotic treatment, genes associated with biofilm formation are stimulated and the bacteria convert to the biofilm phenotype [9]. It is likely that this extract failed to inhibit biofilm formation, but instead induced stress that stimulated the bacteria to switch into the biofilm phenotype from planktonic form. At 50 μg/ml the ethanol extract failed to inhibit *M. smegmatis* biofilm formation but it had a MIC of 12.5 μg/ml. These results suggest that the inhibition of *M. smegmatis* biofilm formation requires a higher concentration of the extract sample than the concentration that is required to inhibit bacterial growth. The different durations of incubation with the extract for each assay could also account for the variations in sensitivity to the extracts observed. In the growth inhibitory assays, *M. smegmatis* was incubated for 24 h in the presence of the extract. In the inhibition of the biofilm formation assay, the mycobacteria were incubated with the extract for 72 h.

When kanamycin was used alone at MIC value, the drug failed to inhibit biofilm formation. When kanamycin, at 0.25 x MIC value was combined with the extract, biofilm formation was then inhibited. It was important to determine if kanamycin had any significant effects on biofilm formation in the combined extract/kanamycin mixture since kanamycin at MIC had failed to inhibit *M. smegmatis* biofilm formation. To further explore this finding, the pattern of biofilm inhibition at varying extract concentration and constant kanamycin concentration at the MIC value were determined. It was noted that 50 μg/ml of the ethanol extract inhibited M*. smegmatis* biofilm formation but had failed to inhibit growth. These results, therefore, suggested a synergistic effect between the extract and kanamycin.

*P. curatellifolia* ethanol leaf extract has been confirmed to contain phytochemicals such as steroids, tannins, flavonoids and saponins [26] and these phytochemicals have been demonstrated to associate with bacterial proteins and inhibit microbial adhesion, enzymes, cell envelop and transport proteins [46–48]. It is, therefore, likely that the ethanol extract inhibited biofilm formation through some of these mechanisms. Bacterial adhesion is important during biofilm formation [49] and agents that disrupt bacterial adhesion to surfaces have the potential to act as anti-biofilm agents.

It was also found that the ethanol extract at a lower concentration of 12.5 μg/ml, when combined with 1.6 μg/ml kanamycin, actually promoted biofilm formation. A possible explanation for this observation is that the extract/kanamycin mixture at that concentration was less effective and did not inhibit biofilm formation. Therefore, *M. smegmatis* were able to form the colonies that transform into biofilms. It is possible that this extract/kanamycin mixture might have induced antibiotic stress in the bacteria [50], resulting in the development of bacilli that were more adapted to the antibiotic/extract mixture and could grow vigorously into biofilms as observed.

## Conclusion

In conclusion*,* our study showed that the leaves from *P. curatellifolia* are a potential source of phytochemicals with activity against the growth and biofilm formation of *M. smegmatis*. The water, DCM and ethanol leaf extracts of *P. curatellifolia* inhibited biofilm formation. Extracts that inhibit *M. smegmatis* growth do not necessary inhibit biofilm formation. Combining the ethanol extract with kanamycin had an effect of enhancing the antimycobacterial effect of the extract. Further studies with purified compounds from *P. curatellifolia* need to be carried out from the ethanol extract, water and DCM extracts in order to isolate phytochemicals that inhibit biofilm formation in *M. smegmatis*. In addition further work needs to be carried out to determine if the same effects are observed with the clinical mycobacterial strain *M. tuberculosis*.
